# High temperatures alter physiological properties of pyramidal cells and inhibitory interneurons in hippocampus

**DOI:** 10.3389/fncel.2012.00027

**Published:** 2012-07-06

**Authors:** Jennifer A. Kim, Barry W. Connors

**Affiliations:** Department of Neuroscience, Brown University, ProvidenceRI, USA

**Keywords:** febrile seizures, hippocampal neurons, hyperthermia, inhibition

## Abstract

Temperature has multiple effects on neurons, yet little is known about the effects of high temperature on the physiology of mammalian central neurons. Hyperthermia can influence behavior and cause febrile seizures. We studied the effects of acute hyperthermia on the immature hippocampus *in vitro* by recording from pyramidal neurons and inhibitory oriens-lacunosum moleculare (O-LM) interneurons (identified by green fluorescent protein (GFP) expression in the GIN mouse line). Warming to 41°C caused depolarization, spontaneous action potentials, reduced input resistance and membrane time constant, and increased spontaneous synaptic activity of most pyramidal cells and O-LM interneurons. Pyramidal neurons of area CA3 were more strongly excited by hyperthermia than those of area CA1. About 90% of O-LM interneurons in both CA1 and CA3 increased their firing rates at hyperthermic temperatures; interneurons in CA3 fired faster than those in CA1 on average. Blockade of fast synaptic transmission did not abolish the effect of hyperthermia on neuronal excitability. Our results suggest that hyperthermia increases hippocampal excitability, particularly in seizure-prone area CA3, by altering the intrinsic membrane properties of pyramidal cells and interneurons.

## Introduction

Temperature is a tightly regulated variable in the mammalian brain. Even small physiologic fluctuations in brain temperature are known to alter neuron physiology and function (Moser et al., 1993; Andersen and Moser, 1995). Effects of subphysiologic temperatures have been carefully studied. Cooling alters intrinsic membrane properties, spike firing rates, and network synchrony of mammalian central neurons (Thompson et al., 1985; Dean and Boulant, 1992; Rubinsky et al., 2007). Small temperature reductions can have strong effects on synaptic vesicle function, vesicle release processes, and presynaptic mechanisms of plasticity (Katz and Miledi, 1965; Andersen and Moser, 1995; Micheva and Smith, 2005). Temperature has complex, non-linear effects on brain tissue, so the physiological effects of cooler temperatures are of limited help in predicting the effects of hyperthermia. Few studies have looked at the acute effects of high temperatures on the physiology of central mammalian neurons. Warming hippocampal slices causes a transient increase in population spike amplitude followed by a decrease when maintained at 37°C (Schiff and Somjen, 1985; Fujii et al., 2002). Propagation of presynaptic action potentials along Schaffer collateral axons also decreases as temperature rises from 32 to 38°C (Takeya et al., 2002). Investigations of temperatures above 37°C have been very limited. One group reported that hyperthermia up to 40°C caused spreading depression in hippocampus *in vitro* (Wu and Fisher, 2000; Wu et al., 2001). Cell metabolism and temperature are closely related. Some experimental effects ascribed to high temperature may have been due to changes in metabolic demands and hypoxia, which alters neuronal physiology (Kawasaki et al., 1990; Jensen et al., 1991, 1992; Jiang and Haddad, 1992; Applegate et al., 1996; Jensen and Wang, 1996; Jensen and Baram, 2000; Sanchez and Jensen, 2005).

High temperatures can have dramatic effects on brain function, and pathologically high temperatures have important clinical consequences. For instance, febrile seizures are the most common type of seizure in infants and young children, affecting 3–5% of the pediatric population (Lowenstein, 2005). These seizures occur during common childhood infections that cause fevers above about 38°C. Experimental models of febrile seizures have demonstrated that seizures can be induced during development by increasing ambient temperature without exposure to infection (Nealis et al., 1978; Holtzman et al., 1981; Morimoto et al., 1991; Tancredi et al., 1992; Ullal et al., 1996; Baram, 2002; Schuchmann et al., 2006). This implies that high temperature *per se* can trigger seizures in the immature brain. Interestingly, new data suggest that high temperatures may have beneficial effects in altering developmentally abnormal neuron physiology. In one report, some children with autism spectrum disorders displayed fewer aberrant behaviors during febrile states (Curran et al., 2007). The cellular mechanisms by which hyperthermic temperatures cause clinical effects are entirely unknown.

Inhibitory synapses are a key component of brain circuitry. Impaired synaptic inhibition has been implicated in the mechanisms of many seizure types (Magloczky and Freund, 2005). Even a small reduction of GABA_A_ receptor-mediated inhibition can lead to seizure initiation and propagation in neocortex (Chagnac-Amitai and Connors, 1989). Experimental studies of the long-term effects of complex febrile seizures have reported both cellular and molecular changes in synaptic inhibition (Chen et al., 1999, 2001; Brewster et al., 2002; Han et al., 2006; Kang et al., 2009). There is some evidence that acute hyperthermia decreases GABA_A_ receptor-mediated synaptic transmission onto CA1 pyramidal neurons *in vitro* (Qu et al., 2007; Qu and Leung, 2008, 2009), although not all studies agree on this point (Hill et al., 2011). Mutations of GABA_A_ receptors are associated with generalized epilepsy with febrile seizures plus (GEFS+) (Lowenstein, 2005; Nakayama and Arinami, 2006; Nakayama, 2009).

Despite the suggestive links between febrile seizures and inhibition, the effects of high temperature on the properties of inhibitory interneurons have not been investigated directly. This task is complicated by the large variety of distinct interneuron types that populate the cerebral cortex (Klausberger and Somogyi, 2008). The use of mouse lines that express green fluorescent protein (GFP) in specific subtypes of interneurons has been very helpful. One well-studied transgenic line, the GIN mouse, expresses GFP selectively in a subset of somatostatin-expressing interneurons (Oliva et al., 2000). In the hippocampus, these cells were identified as primarily oriens-lacunosum moleculare (O-LM) interneurons. O-LM interneurons selectively target the distal dendrites of pyramidal cells. CA1 pyramidal cells provide the main excitatory inputs to O-LM cells, and these serve as a part of a critical feedback circuit onto both pyramidal cells and other interneurons (Blasco-Ibanez and Freund, 1995; Pouille and Scanziani, 2004).

Here we used C57BL/6 and GIN mouse lines to test the acute effects of hyperthermic temperatures on pyramidal cells and interneurons of the hippocampus *in vitro*. We focused on changes of intrinsic membrane properties, neuronal excitability, and spontaneous excitatory and inhibitory synaptic events. Our data imply that hyperthermic temperatures increase the excitability of both excitatory and inhibitory neurons by effects on their intrinsic membrane properties. There were both region- and cell type-specific differences in heat sensitivity. These results suggest that the effects of hyperthermia on neuron populations are not uniform, but rather may preferentially excite more epileptogenic regions of cortex.

## Methods

### Tissue preparation and recording procedures

All procedures were approved by the Brown University Institutional Animal Care and Use Committee. Wild-type C57BL/6 mice, originally obtained from Charles River Inc. and bred in-house, were used for all principal cell recordings. GIN mice (Oliva et al., 2000), originally obtained from Jackson Labs (Bar Harbor, ME) and maintained in-house on an FvB background strain, were used for interneuron recordings described here. Coronal slices, 350 μm thick, were prepared from P13 to 16 mice using slicing procedures similar to those previously described (Cruikshank et al., 2007). This slice thickness was chosen to allow sufficient oxygen diffusion while minimally disrupting network connectivity and dendritic arbors. Recordings were made from dorsal hippocampus cut in a plane that was roughly transverse to the long axis of the structure. Slices were kept in a submerged-style holding chamber at 32°C for at least 30 min and maintained at room temperature until transferred to a submerged recording chamber. Artificial cerebrospinal fluid (ACSF) contained (in mM): 126 NaCl, 3 KCl, 1.25 NaH_2_PO_4_, 2 MgSO_4_, 26 NaHCO_3_, 10 dextrose, and 2 CaCl_2_, saturated with 95% O_2_/5% CO_2_.

Whole-cell current-clamp recordings were made from visually identified pyramidal neurons, dentate granule cells, and GFP-expressing stratum oriens interneurons in areas CA1 and CA3 using micropipettes (3–6 MΩ) containing (in mM): 130 potassium gluconate, 4 KCl, 2 NaCl, 10 HEPES, 0.2 EGTA, 4 ATP-Mg, 0.3 GTP-Tris, 14 phosphocreatine-Tris (pH = 7.25, ~290 mOsm). Hippocampal CA1 and CA3 pyramidal cells were visualized with infrared differential interference contrast (IR-DIC) optics, and recordings were made from somata. Hippocampal GFP-expressing (GFP+) CA1 and CA3 stratum oriens interneurons were first located using epifluorescence illumination. Once identified, these cells were then targeted with microelectrodes under IR-DIC optics.

All recordings were acquired using Axon Instruments hardware (Axoprobe 1A) and a LabView program developed in-house (Jay R. Gibson and Kristen A. Richardson). Data were sampled at 20 kHz and low-pass filtered for analyses at 3 kHz. The electrode resistance was monitored and compensated online throughout the experiment. Resting potentials were measured within 2 min of membrane break-in and steady-state potentials were usually adjusted to the mean population resting potential (CA1 pyramidal −60 to −61 mV, CA3 pyramidal −65 to −66 mV, DG −67 to −68 mV, CA1 GIN −57 to −58 mV, CA3 GIN cells −55 to −56 mV). Membrane potentials reported here were not corrected for the liquid junction potential.

Perfusate temperature for all *in vitro* experiments was regulated by a TC-324A temperature controller and an in-line heater (Warner Instruments). The actual temperature of the perfusate was monitored via thermistor probes placed near the slice. To determine the temperature responses of hippocampal pyramidal cells, the perfusate temperature was increased from 30°C to approximately 41°C over 120 s (~0.1 °C/s) while recording membrane potential. The rate of temperature rise and the duration of hyperthermia may be important when modeling febrile seizures (Thon et al., 2002). Thus, we used both a rate and duration of hyperthermia that mimicked *in vivo* animal models (Tancredi et al., 1992; Baram et al., 1997). The relatively rapid temperature jumps were also intended to minimize effects from temperature-dependent changes in metabolic rate. Mice at the developmental age of P14 have a basal body temperature of approximately 32°C. We made recordings from neurons at 32 and 30°C with no difference in baseline activity observed (data unpublished). We maintained ACSF flow rates ~3 ml/min for efficient heating/cooling and to minimize chances of hypoxia.

### Data analysis

Data analysis programs were written in Matlab (Jennifer A. Kim and Kristen A. Richardson). Membrane time-constants (τ_*m*_) and input resistances (*R*_in_) were calculated from voltage responses to small negative current pulses (typically −20 pA, 600 ms). To estimate τ_*m*_ the voltage responses were fitted with single exponentials, skipping the initial 3 ms after the start of the stimulus pulse and ending before any voltage “sag” became apparent. All combined data are expressed as mean ± standard error of the mean (S.E.M.). Increases in spiking, bursting and/or depolarization block in response to heating were identified manually. The criterion for increased spiking was any significant increase in action potential activity during the rise or maintenance of high temperature. Bursting was defined as two or more spikes in rapid succession without significant repolarization in between spikes. Depolarization block was defined as significant depolarization accompanied by abrupt termination of spontaneous spiking. Rates of increased spiking, bursting and/or depolarization block in response to heat were compared using the two-tailed Fisher's exact test. Comparisons of the means observed in different pyramidal populations were performed using two-tailed, unpaired *t*-tests whereas comparisons within cell populations at 30 vs. 41°C were performed using two-tailed, paired *t*-tests. Significance was defined as *p* < 0.05. The *n*-values reported refer to the total number of cells recorded (usually one cell per slice, and occasionally two). Spike rates were determined by calculating the inverse of interspike intervals averaged over 5 s. Temperature measures were also made during each 5 s period and rounded to the closest whole °C to obtain mean spike rates per °C.

To measure the strength of electrical coupling between pairs of GIN cells, hyperpolarizing current steps of 600 ms duration were applied to each cell in succession. Coupling coefficients (CCs) were calculated as the ratio of voltage change in the non-injected cell to the voltage change in the injected cell (Connors and Long, 2004).

### Drugs

Kynurenic acid and picrotoxin were purchased from Sigma-Aldrich. Picrotoxin was dissolved in DMSO and then diluted to a final concentration of 100 μM in ACSF. Kynurenic acid was measured and added to ACSF at the beginning of each experiment day for a final concentration of 2 mM.

## Results

### Hyperthermic temperatures excite pyramidal cells and dentate granule cells

**Figure 1:**
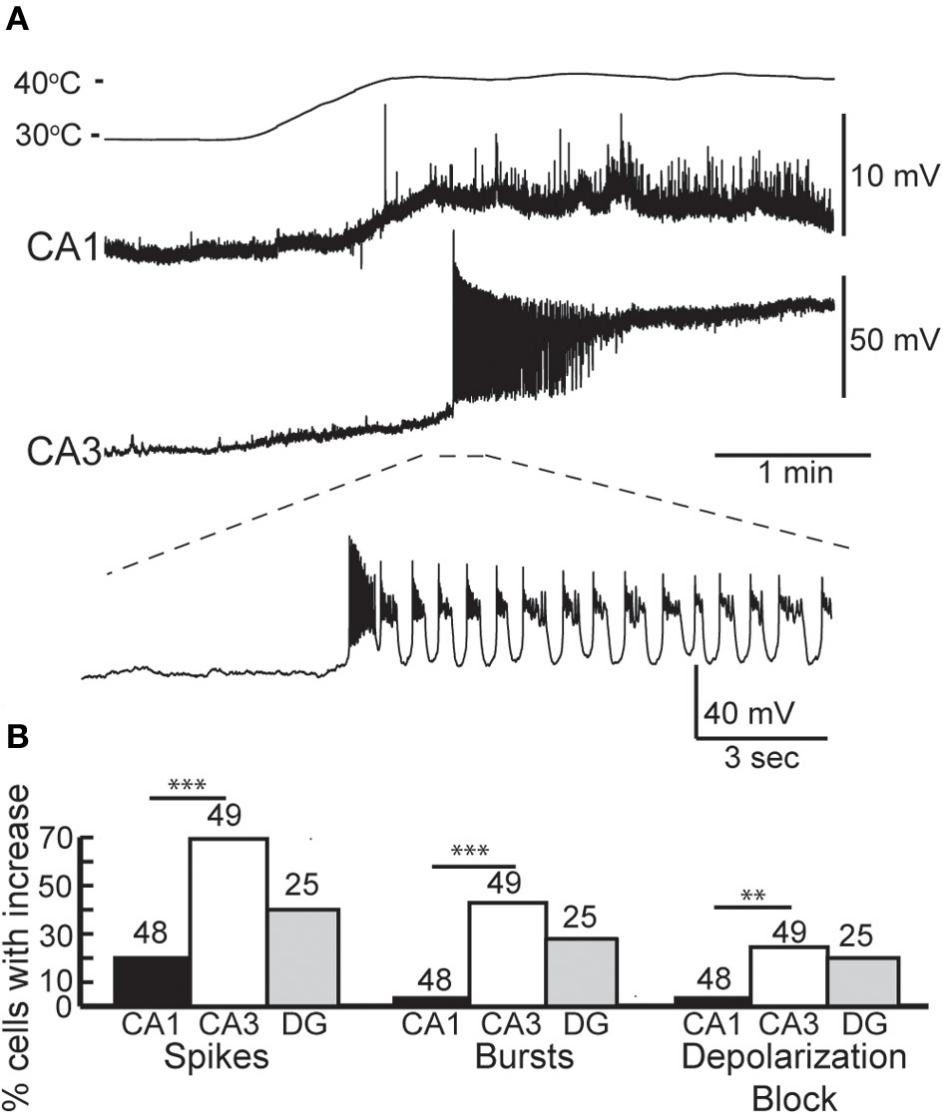
**Effects of hyperthermia on CA1 and CA3 pyramidal cells. (A)** Representative recordings from CA1 and CA3 neurons; note difference in voltage scale. Lowest trace shows initial phase of hyperthermia-induced spiking and bursting in CA3 cell. **(B)** Hyperthermia increased spiking, bursting, and depolarization block most often in CA3 pyramidal cells. Total number of cells listed above each bar. ^**^p < 0.01; ^***^p < 0.005 (Fisher's exact test).

We made whole-cell recordings from principal neurons in CA1 and CA3 while increasing the temperature of the perfusion solution from 30 to 41°C. Hyperthermia-induced depolarization and spiking in both cell types, although CA3 cells tended to be most sensitive (Figure 1A). Hyperthermia-induced increases in spiking differed between regions: about 69% of CA3 pyramidal cells and 21% of CA1 cells fired in response to heat ramps (Figure 1B). Burst-firing was also more commonly induced by hyperthermia in CA3 than in CA1 (Figure 1B). Furthermore, during heating some of the cells reached a membrane potential positive enough to cause depolarization block, presumably caused by sodium channel inactivation (see CA3 cell in Figure 1A); this also occurred most frequently in CA3 pyramidal cells (Figure 1B).

We tested whether neurons recovered from short, 5 min exposures to hyperthermia by returning the ACSF to 30°C. Recovery during this cooling period was quite variable. The membranes of some neurons fully repolarized and the spiking initiated during hyperthermia ceased upon return to the control temperature. Full recovery was more common in CA1 pyramidal cells (CA1 = 46%, CA3 = 16%), perhaps due to their lower rates of hyperthermia-induced spiking and depolarization block. Other cells only partially repolarized or slowed, but did not cease spiking; partial recovery was most common in CA3 pyramidal cells (CA1 = 12.5%, CA3 = 47%). Some cells did not repolarize or reduce their firing rates during cooling (CA1 = 19%, CA3 = 33%).

**Figure 2:**
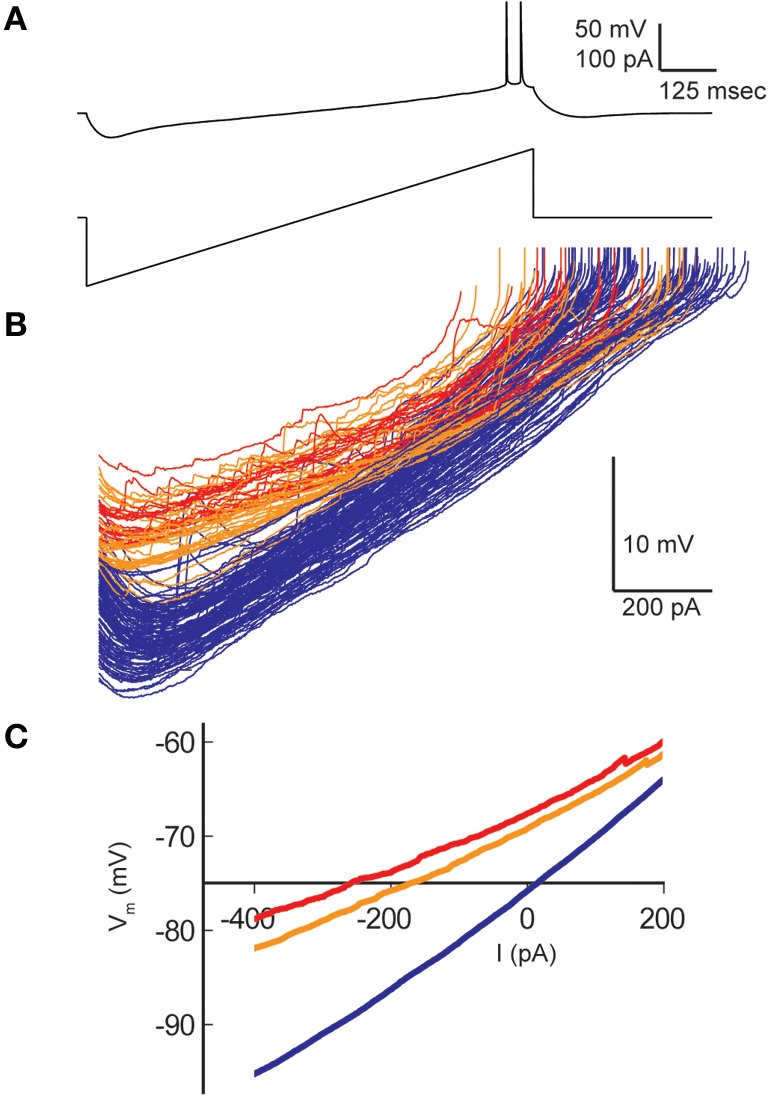
**Effect of hyperthermia on current-voltage relationship of CA1 pyramidal cells. (A)** Ramp currents were injected into CA1 pyramidal cells every 5 s and voltage responses were measured. **(B)** Current-voltage plots coded by temperature: < 031°C (blue), 31–38°C (orange), > 38°C (red). **(C)** Means of the current-voltage plots for each temperature category, truncated at spike initiation.

Resting membrane potentials depolarized in both pyramidal cell types during heating (CA1, 8.2 mV; CA3, 14.4 mV). To estimate neuronal input resistances, we injected small hyperpolarizing current steps every 5 s during the temperature protocol. In CA1 cells, input resistance decreased significantly from a mean of 208 MΩ at 30°C to 167 MΩ at 41°C. The input resistance of CA3 cells fell from 358 MΩ to 271 MΩ and τ_*m*_ decreased from 57 to 46 ms over this temperature range (Table 1). τ_*m*_ was not significantly altered at high temperature in CA1 pyramidal cells (30°C, 20 msec; 41°C, 22 ms). However, the time-constants of these cell populations can be difficult to measure because non-passive currents are so easily activated (Spruston and McBain, 2007). To provide a more complete measure of the current-voltage relationship during heating, we injected 1 s ramp currents into pyramidal cells. Current amplitudes were adjusted in each cell to induce a voltage excursion of about 30 mV centered on resting potential, and measurements were made at temperatures between 30°C and 41°C (Figure 2A; CA1: *n* = 4, CA3: *n* = 7). These measurements showed that membrane depolarization *per se* did not induce the decreased input resistance and τ_*m*_ we observed during high temperatures (Figures 2B,C). In fact, at 30°C depolarization alone increased the slope of the current-voltage curve, presumably due to the opening of voltage-dependent cation conductances (Hotson et al., 1979).

**Table 1:** **Passive membrane properties of principal cells at hyperthermic temperatures**.

	**CA1 pyramidal cells at 30°C**	**CA1 pyramidal cells at 41°C**	**CA3 pyramidal cells at 30°C**	**CA3 pyramidal cells at 41°C**	**Dentate granule cells at 30°C**	**Dentate granule cells at 41°C**
*V*_*m*_ (mV)	−63.3 ± 0.9	−55.1 ± 2.4^***^	−62.1 ± 0.7	−47.7 ± 1.7^***^	−67.3 ± 1.8	−52.6 ± 2.4^*^
*R*_*in*_ (MΩ)	208 ± 24	167 ± 22^***^	358 ± 19	271 ± 24^***^	987 ± 118	788 ± 91^***^
τ_*m*_ (msec)	19.9 ± 1.3	21.9 ± 1.9	57.30 ± 2.5	45.7 ± 1.9^**^	34.8 ± 2.8	34.7 ± 1.5

All data are mean ± S.E.M; CA1 pyramidal (n = 19), CA3 pyramidal (n = 38), DG (n = 19);

* p < 0.05,

** p < 0.01,

*** p < 0.005. V_m_: membrane potential, R_in_: input resistance, τ_m_: time-constant.

Dentate granule cells comprise a third major group of excitatory principal cells in the hippocampus (Shepherd, 2004). Hyperthermia-induced an increase in spiking in 40% of the dentate granule cells we recorded (*n* = 25; Figure 1B), which was intermediate between CA1 and CA3 effects. Heat ramps also depolarized granule cells and decreased their input resistance (Table 1), as observed in CA1 and CA3 pyramidal cells; membrane time-constants of granule cells, however, were unchanged by high temperature.

### Hyperthermia increases synaptic activity in principal neurons

**Figure 3:**
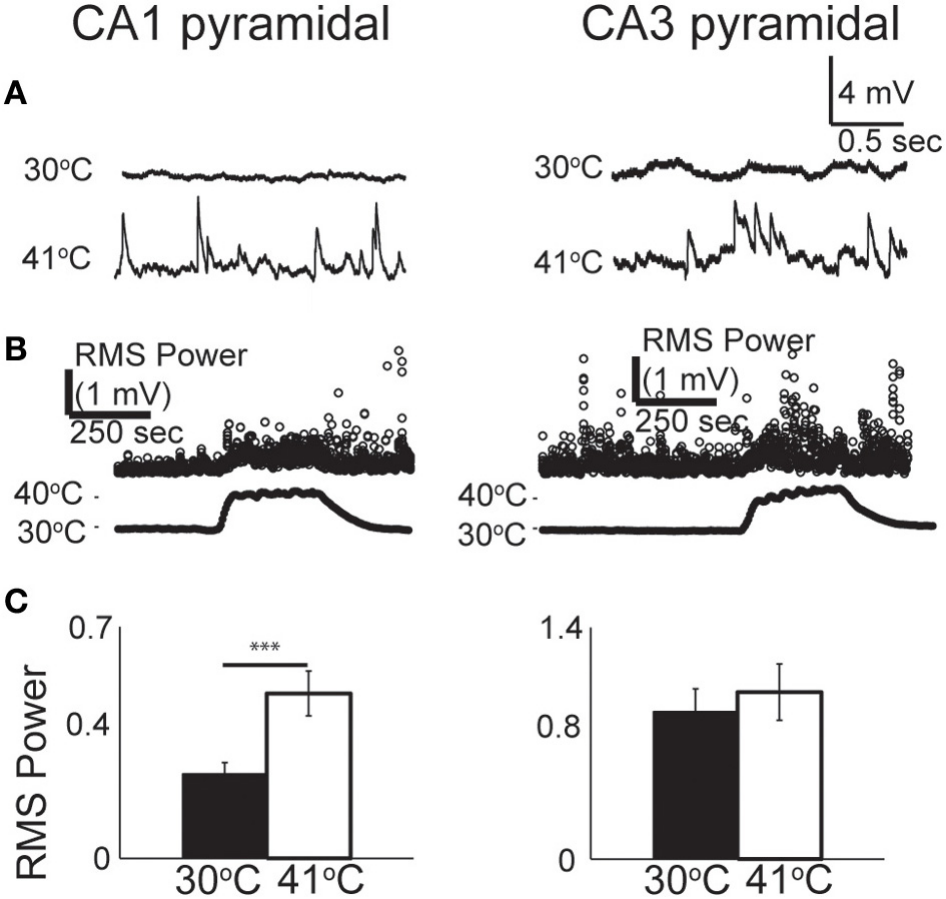
**Increase in spontaneous synaptic activity during hyperthermia. (A)** Example voltage traces from CA1 and CA3 pyramidal cells at control and hyperthermic temperatures. **(B)** Example time-course of RMS measurements during period of hyperthermia. **(C)** Bar graphs showing mean and S.E.M. calculations of RMS at 30 and 41°C. Paired, two-tailed *t*-tests used to compare changes. ^***^p < 0.005.

From our observations of the raw voltage traces, it was clear that increases in synaptic activity were also induced by hyperthermia (Figure 3A). In current-clamp we could not precisely separate synaptic changes into EPSPs and IPSPs, so we used a root mean squared (RMS) calculation of resting membrane potential to estimate the overall change in synaptic activity (Figure 3B). RMS increased in both CA1 and CA3 pyramidal cells as temperature rose to febrile levels. Because heat-induced firing occurred so frequently in CA3 cells, calculations of the changes in RMS were often difficult to quantify; nevertheless, increases in synaptic activity were observed qualitatively in most CA3 cells. RMS changes were statistically significant in CA1 (Figure 3C).

To test the role of synaptic inputs on cell excitability, we blocked ionotropic glutamate and GABA_A_ receptors by bathing neurons in 2 mM kynurenic acid and 100 μM picrotoxin for 15 min prior to heating. The drug combination eliminated spontaneous PSPs, as determined by the reduction in RMS, however, it did not change the likelihood of hyperthermia-induced spike firing in either CA1 (21 vs 15%, *n* = 20) or CA3 pyramidal cells (69 vs 73%, *n* = 26). The effects of hyperthermia on input resistance and membrane time constant were also not significantly altered by the receptor antagonists (data not shown). We conclude that heat-induced changes in spiking or intrinsic membrane properties were not secondary to changes in spontaneous fast synaptic activity.

### GIN inhibitory interneurons are particularly sensitive to febrile temperatures

**Figure 4:**
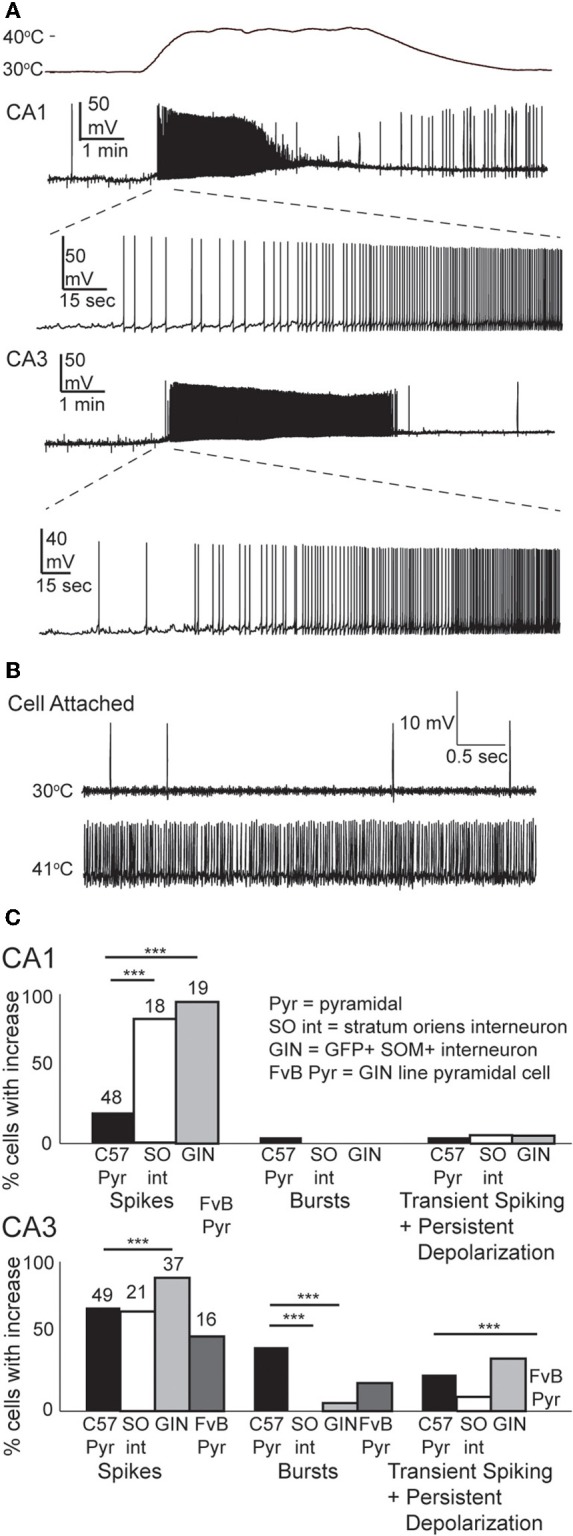
**Hyperthermia excites GIN interneurons. (A)** Whole-cell recordings from GIN cells in areas CA1 and CA3. Initial phase of hyperthermia-induced firing in both cells is magnified to show individual action potentials. **(B)** Recording from a CA3 GIN cell in cell-attached mode. Firing pattern is similar to that observed using whole-cell recording. **(C)** Bars plot the fraction of neurons of each type that showed increased spikes, bursts, and transient spiking plus persistent depolarization. ^**^p < 0.01; ^***^p < 0.005 (Fisher's exact test).

We tested the effects of hyperthermia on a set of easily identified, somatostatin-positive, GFP-expressing inhibitory interneurons in GIN mice; we will refer to these as “GIN cells.” The somata of GIN cells were located in the stratum oriens, and most of them are known to be O-LM interneurons (Oliva et al., 2000). Hyperthermia induced depolarization and spike firing in GIN neurons from both CA1 and CA3 (Figure 4A). To ensure that this result was not an artifact of the whole-cell recording technique, we recorded some GIN cells in cell-attached mode. The spiking results from cell-attached recordings were very similar to those obtained with the whole-cell technique (*n* = 4; Figure 4B).

**Figure 5:**
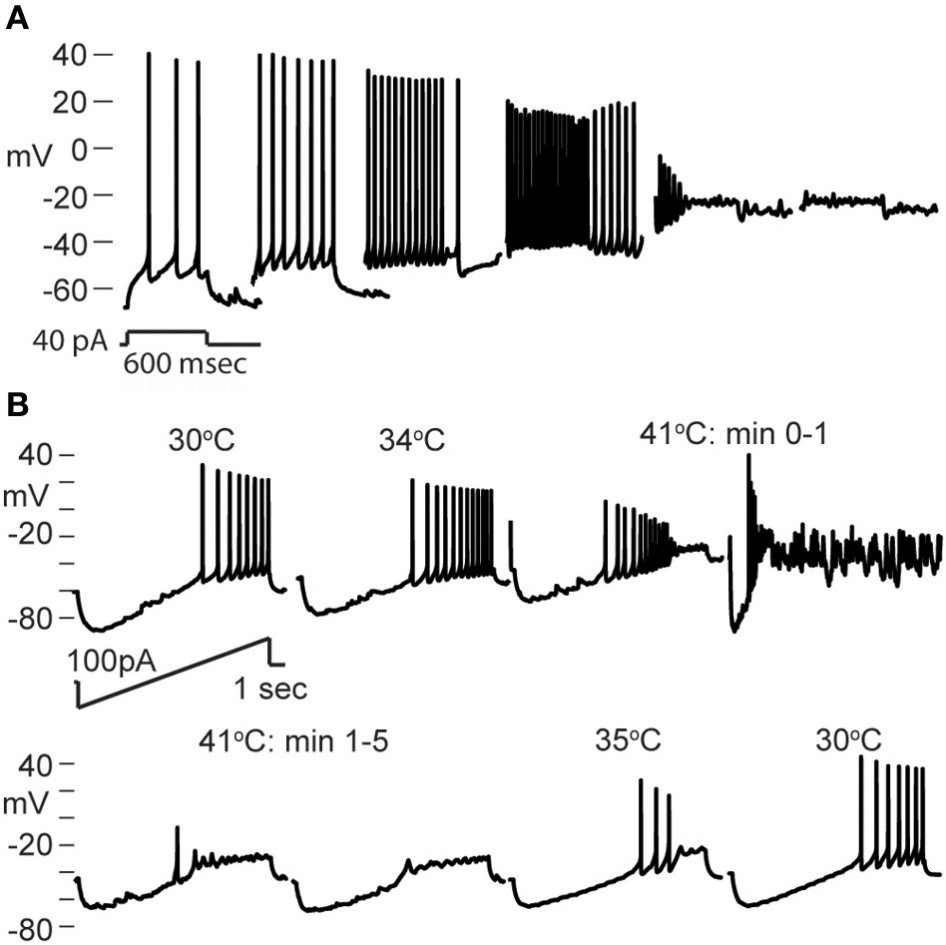
**Effect of depolarization and hyperthermia on spike generation. (A)** CA3 GIN cell injected with +40 pA to induce spiking at various starting membrane potentials at 30°C. Spikes were generated until about −25 mV. **(B)** CA3 GIN cell injected with ±100 pA ramp currents (1 s duration) periodically during hyperthermia protocol. At 41°C, spikes were eliminated even when resting potential was only about −50 mV. When temperature was reduced, the cell recovered its spiking ability.

Nearly all GIN interneurons in CA1 and CA3 spiked rapidly in response to hyperthermia (Figure 4C). Some GIN cells fired spontaneously at a basal rate upon break-in at 30°C; such cells were only considered to show hyperthermia-induced spiking if their rate increased significantly above the basal rate. Hyperthermia-induced burst-firing was rare in hippocampal GIN cells (CA1 = 0%; CA3 = 5.4%). Some GIN cells showed transient spiking during hyperthermia, then ceased firing (Figure 4A, CA1 cell), whereas others spiked for the entire five min period of hyperthermia. Transient spiking with persistent depolarization occurred in <10% of CA1 GIN cells and about 35% of CA3 GIN cells. At 30°C, hippocampal GIN cells were entirely capable of generating action potentials at −40 mV and did not cease firing until depolarized to about −30 mV (Figure 5A). Notably, some GIN cells stopped firing at high temperature despite membrane potentials of −40 mV. One second ramp stimulus currents were used to test spike thresholds during hyperthermia (Figure 5B). At 30°C, these ramp currents induced a sufficiently large voltage change to induce firing. Higher temperatures induced spikes sooner along the ramp, but after > 1 min at 41°C the cell no longer spiked even at resting potentials around −40 mV. Cooling back to 30°C led to a return of control spiking behavior, implying that GIN cells were not irreversibly damaged by the 5 min exposure to hyperthermia. These data show that hyperthermia initially increases the excitability of most GIN interneurons, and that depolarization alone cannot explain the sustained hyperthermia-induced blockade of spiking in some GIN cells.

**Figure 6:**
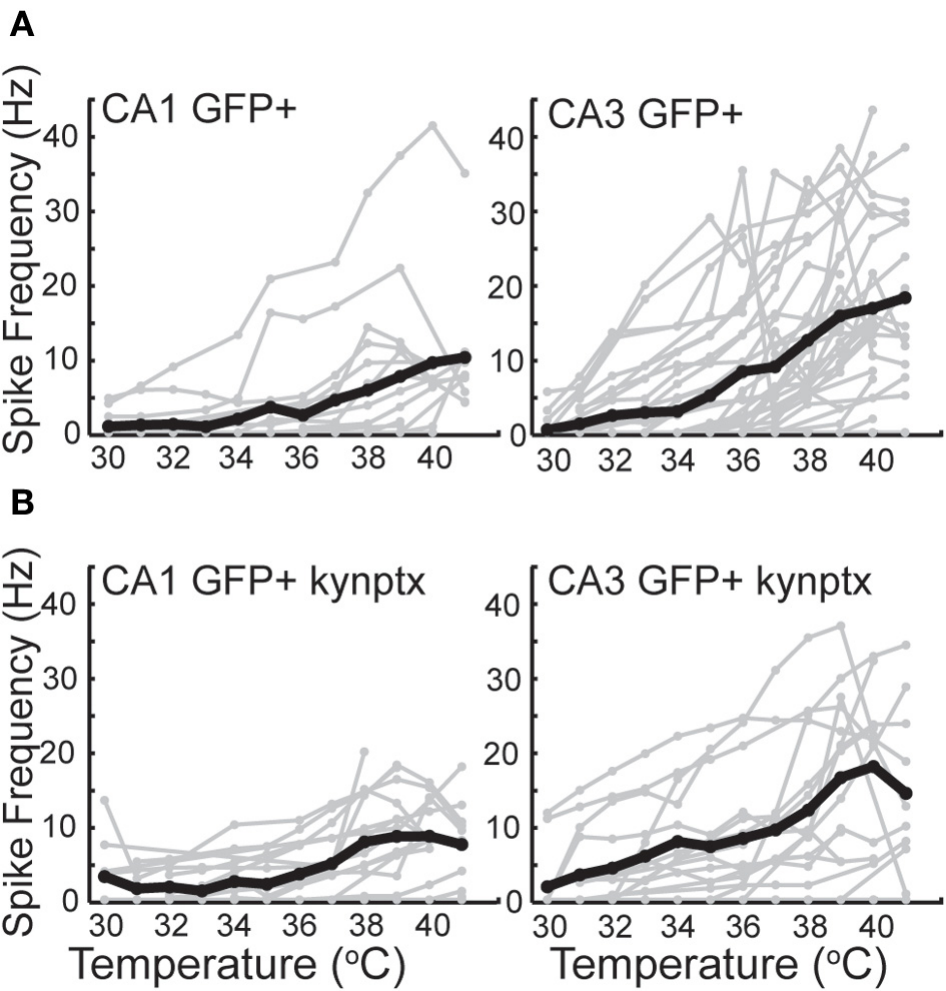
**Spike rate increases greater in CA3 GIN cells than CA1 GIN from 30°C to 42°C. (A)** In normal ACSF. **(B)** In the presence of kynurenic acid and picrotoxin. Individual cells are plotted in gray and averages are plotted in black.

GIN interneurons in the neocortex can be activated by a variety of conditions, with preferred spontaneous firing rates in the theta frequency range, 3–11 Hz (Fanselow et al., 2008). Consistent with this, we found that GIN cells in CA1 reached a maximal average frequency of around 10 Hz at 41°C (Figure 6A). GIN cells in CA3, however, reached an average of 20 Hz at 41°C (Figure 6A). The difference between CA1 and CA3 was significant (*p* = 0.02). These spike rates were not dependent on fast synaptic transmission in either hippocampal region (Figure 6B). As with pyramidal cells, hyperthermia significantly decreased the input resistance and τ_*m*_ of GIN interneurons (Table 2).

**Table 2:** **Passive membrane properties of GIN cells at hyperthermic temperatures**.

	**CA1 GIN cell at 30°C**	**CA1 GIN cell at 41°C**	**CA3 GIN cell at 30°C**	**CA3 GIN cell at 41°C**
*V*_*m*_ (mV)	−57.0 ± 0.7	−44.7 ± 1.1^***^	−55.6 ± 0.8	−44.0 ± 1.1^***^
*R*_*in*_ (MΩ)	565 ± 77	410 ± 76^**^	322 ± 28	221 ± 25^***^
τ_*m*_ (ms)	51.4 ± 2.8	35.9 ± 3.6^***^	42.7 ± 2.4	36.5 ± 1.9^*^

All data are mean + S.E.M; CA1 GIN (n = 14), CA3 GIN (n = 34);

* p < 0.05,

** p < 0.01,

*** p < 0.005.

Our results suggest that GIN interneurons are more sensitive than pyramidal cells to febrile temperatures. One potential confound is the different genetic background of the mice used for most of our pyramidal cell and GIN cell experiments (Crawley, 2000). However, when we tested the excitability of CA3 pyramidal cells from the GIN line of mice, with their FvB background, they showed lower sensitivity to heating than CA3 pyramidal cells from C57BL/6 mice (Figure 4C). Strain differences do not seem to account for the differences between pyramidal cells and GIN interneurons.

### Hyperthermia increased synaptic activity in GIN interneurons

**Figure 7:**
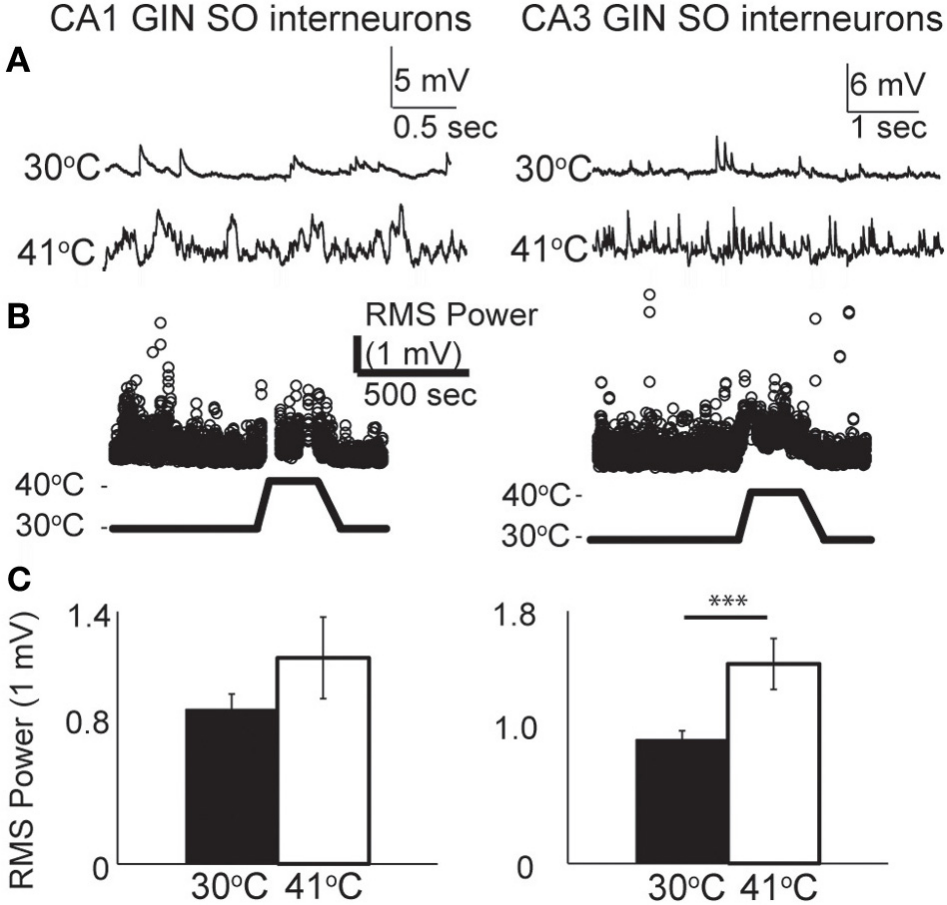
**Hyperthermia increased spontaneous synaptic activity. (A)** Example voltage traces of CA1 and CA3 GIN cells showing increased synaptic events during hyperthermia. **(B)** Example time-course of RMS measurement during hyperthermia. **(C)** Bars show mean ± S.E.M. of RMS at 30 and 41°C. Paired, two-tailed *t*-tests were used to compare changes; ^***^p < 0.005.

As with pyramidal cells, there was a noticeable increase in spontaneous synaptic activity in GIN cells during hyperthermia (Figure 7A). Synaptic activity was estimated by the RMS of membrane potential, and trials with significant spiking were discarded. RMS increased with temperature in both CA1 and CA3 GIN cells (Figures 7B,C), although the change was only statistically significant in CA3. We tested whether synaptic activity contributed significantly to the increase in cell excitability induced by hyperthermia by adding 2 mM kynurenic acid and 100 μM. The probability of spiking during hyperthermia was unchanged in the drug-treated cells (CA1: 95 vs 79%, *n* = 19; CA3: 89 vs 89%, *n* = 19), compared to control cells. Furthermore, in the presence of the receptor blockers the effects of hyperthermia on membrane potential, input resistance, and τ_*m*_ were similar to those in the absence of drugs (data not shown). Maximal spike rates at 41°C were also unaffected by the blockers (Figure 6B). These data suggest that the hyperthermia-induced excitability increases we observed in GIN cells did not depend on changes in spontaneous synaptic activity.

### Hyperthermia may not affect all interneuron subtypes equally

**Figure 8:**
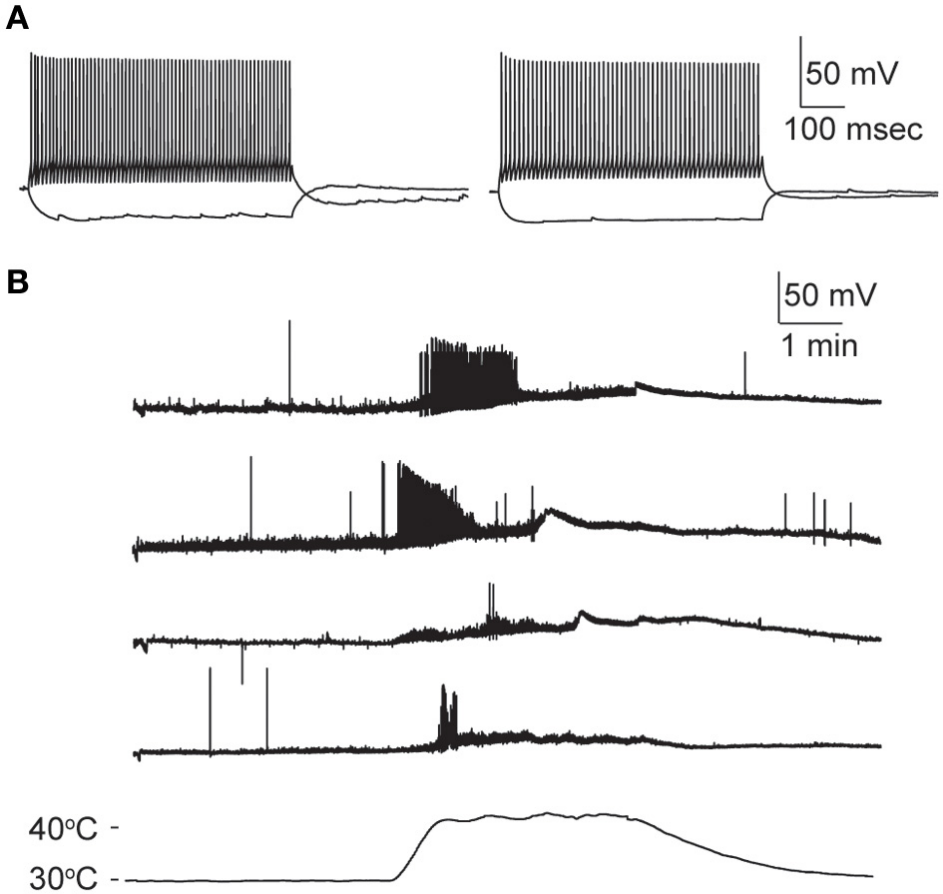
**Hyperthermia increases excitability of fast-spiking non-GIN interneurons of CA3. (A)** Two examples of non-GIN interneurons stimulated with positive and negative current steps. **(B)** Responses of four non-GIN CA3 cells with fast-spiking properties. Temperature time-course plotted in bottom trace.

How does hyperthermia affect non-GIN interneurons in hippocampus? Although it was not the focus of our study, we did a few preliminary recordings of GFP-negative interneurons in stratum oriens of CA1 and CA3. Collectively, the overall sensitivity of these non-GIN cells to hyperthermia was not different from that of GIN cells. Classifying hippocampal interneurons solely with electophysiologic properties is notoriously difficult (Soltesz, 2006). Several of our non-GIN cell sample resembled a “fast-spiking” (FS) interneuron profile (Cesare et al., 1996; Soltesz, 2006; Hu et al., 2010), including brief spike half-widths (<0.7 ms) and minimal spike frequency adaptation during long step current stimuli (> 0.85 frequency adaptation ratio). We identified four CA1 and four CA3 FS interneurons. The temperature sensitivity of the FS cells from CA1 did not differ from that of the GIN cells from CA1. However, none of the FS cells in CA3 fired persistently during heating, contrary to the most common pattern of GIN cells in CA3. One FS cell from CA3 did not spike at all during hyperthermia, and the remaining three spiked only at the beginning of heating (Figure 8). These data imply that heat sensitivity may vary by interneuron subtype and hippocampal subregion.

### Gap junction coupling strength may strengthen during hyperthermia

**Figure 9:**
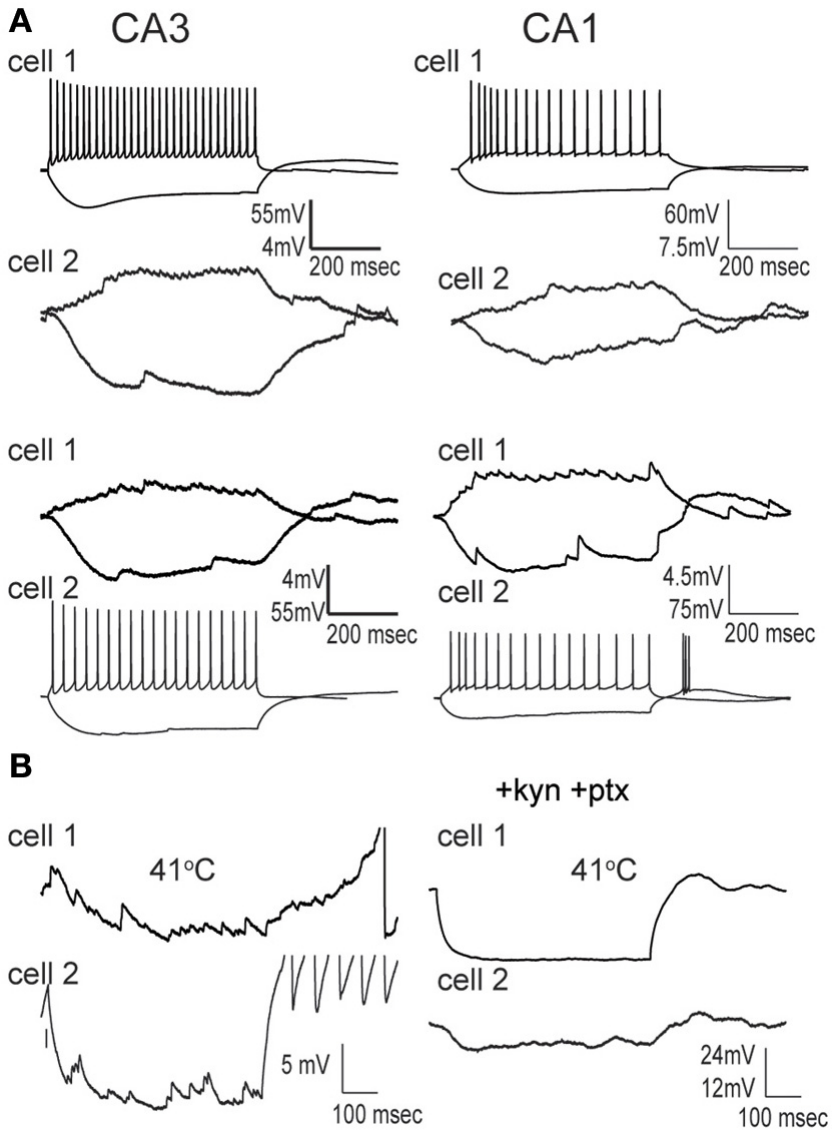
**Gap junction coupling between pairs of GIN cells is preserved at hyperthermic temperature. (A)** Simultaneously recorded pairs of GIN cells in CA3 and CA1 at 30°C exhibit symmetrical electrical coupling during positive and negative current steps injected sequentially into each cell. **(B)** At 41°C, electrical coupling is still measurable across the same cell pairs.

Hippocampal interneurons are electrically coupled by gap junctions, and these connections can mediate considerable interneuron synchrony (Fukuda and Kosaka, 2000; Hormuzdi et al., 2001; Meyer et al., 2002; Connors and Long, 2004; Zhang et al., 2004). The sensitivity of electrical coupling to hyperthermia is unknown. Although rare, in this study we encountered three pairs of GIN cells that were coupled (Figure 9). The effect of hyperthermia was tested on one pair in CA3 in normal ACSF; its average CC was 0.16 at 30°C and increased to 0.32 at 41°C. Another pair of CA3 GIN cells was tested in the presence of kynurenic acid and picrotoxin; it had a CC of 0.10 at 30°C, which increased to 0.26 at 41°C. A pair of GIN interneurons from CA1 was also recorded with blockers; its coupling strength did not change with heating (CC = 0.24 at 30°C; CC = 0.23 at 41°C). Thus, gap junction coupling occurs between GIN interneurons and it is maintained, if not strengthened, at hyperthermic temperatures.

## Discussion

Our results show that hyperthermia increases the intrinsic excitability of both excitatory and inhibitory neurons of the hippocampus. Hyperthermia strongly affected the physiology of all three major excitatory neuron populations. Hyperthermia-induced spiking was most prominent in CA3 pyramidal cells, which usually exhibited a burst-firing pattern. The hypersensitivity of CA3 is consistent with previous studies of other types of seizure models, which demonstrated that CA3 pyramidal cells are particularly susceptible to epileptiform activity (Schwartzkroin and Prince, 1977; Traub and Wong, 1982; Wong and Traub, 1983; Tancredi et al., 1990; Jensen and Yaari, 1997; Rutecki and Yang, 1998; Derchansky et al., 2006; Isaev et al., 2007). Studies of the long-term effects of hyperthermic seizures have focused on the CA1 area (Chen et al., 1999, 2001; Dube et al., 2000; Brewster et al., 2002; Han et al., 2006); post-febrile seizure modifications induced in CA3 should also be explored.

GIN (O-LM) inhibitory interneurons were even more sensitive to febrile temperatures than pyramidal cells. The interneurons robustly fired spikes in response to heat in both CA1 and CA3. Unlike pyramidal cells, GIN cells in CA1 were as likely to generate hyperthermia-induced spikes as those in CA3, although the maximal firing frequency reached at 41°C in CA3 cells was twice as high in that in CA1 cells. O-LM cells are known to preferentially fire in the theta frequency range (3–11 Hz), both *in vivo* and *in vitro* (Fanselow et al., 2008; Klausberger and Somogyi, 2008). O-LM cells are thought to be important for generating and maintaining theta oscillations in the hippocampus (Gloveli et al., 2005). Cortical oscillations and seizures have a close relationship. Cortical oscillation frequencies can change as a result of seizures, such as a change from theta to gamma frequencies after temporal lobe seizure induction (Dugladze et al., 2007), and seizures can also result from changes in cortical oscillations frequencies, such as from sleep spindle (6–14 Hz) to absence seizure (3–4 Hz) frequencies or (Blumenfeld and McCormick, 2000). Hyperthermia may similarly increase the firing frequency of O-LM cells, thereby affecting synchronization states within the hippocampus. Previous reports have shown that when O-LM cells increase their firing frequency, there appears to be an increase in gamma coherence across large distances (Tort et al., 2007). In turn, this may encourage hypersynchrony and facilitate the development of seizures.

Significant changes of intrinsic membrane properties accompanied hyperthermia-induced increases in spiking activity. Membrane potential depolarized and input resistance decreased in all cell types. Previous reports indicate that depolarization of cortical pyramidal cells increases apparent input resistance, as measured in current-clamp, due to the activation of persistent sodium conductances (Connors et al., 1982; Stafstrom et al., 1985; Spruston and Johnston, 1992; Cruikshank et al., 2007). Indeed, we found that depolarization alone, at 30°C, increases input resistance. Thus, if anything, we are probably underestimating the degree to which input resistance is reduced by high temperatures. Another surprising finding is that depolarization block of GIN interneurons occurred at more hyperpolarized potentials during hyperthermia compared to 30°C. These data imply that the mechanism by which hyperthermia leads to depolarization is probably distinct from its mechanisms of altering input resistance and intrinsic excitability.

Our results suggest that studies targeting CA3 populations of neurons may provide the most insight into the mechanisms of heat-induced excitability, which we did not address here. Temperature affects all biochemical processes. Input resistance decreased during hyperthermia in principal cells as well as O-LM interneurons. The combination of depolarization and decreased input resistance suggests that hyperthermia increases membrane sodium and/or calcium conductance. Heat-activated channels, such as transient receptor potential vanilloid 1 (TRPV1) receptors (Dhaka et al., 2006; Gibson et al., 2008), could influence excitability. The effects of interleukin-1β application on different cell types or recordings of mice with an IL-1ra deletion, previously shown to affect febrile seizure threshold would be interesting to investigate as well. Mutations of GABA_A_ and Na^+^ channels can lead to familial forms of generalized epilepsy with complex febrile seizures [GEFS+; (Scheffer and Berkovic, 1997; Spampanato et al., 2004; Nakayama, 2009)]. Febrile seizures lead to long-term changes in hyperpolarization-activated cyclic nucleotide-gated (HCN) channel and endocannabinoid channel signaling (Chen et al., 2001, 2003; Brewster et al., 2002); these processes might also be important in the generation of febrile seizures. A recent study identified a temperature sensitive Na^+^ channel, Nav1.2, that is specifically expressed in the axon initial segment of neurons and may contribute to febrile seizure mechanisms (Thomas et al., 2009). Nav1.1 channels, mutated in some cases of severe myoclonic epilepsy of infancy (Oakley et al., 2009), would also be interesting to explore.

We also observed changes in synaptic activity during hyperthermia. CA3 pyramidal cells have recurrent excitatory synapses (Amaral et al., 1990; Ishizuka et al., 1990; Li et al., 1994; Bains et al., 1999; McIntyre and Schwartzkroin, 2008), so enhanced glutamate release might be particularly important for seizures generated there. Synaptic activity in GIN cells was noticeably increased at hyperthermic temperatures in both CA1 and CA3. However, we found that experimental blockade of ionotropic glutamate and GABA receptor-mediated transmission did not significantly alter the rates of hyperthermia-induced increases in spontaneous spiking. This does not mean that synaptic activity and connectivity are unnecessary for the generation of febrile seizures, but it does suggest that the high temperature increases the intrinsic excitability of pyramidal cells and interneurons.

High temperatures can increase tissue metabolic rate and decrease oxygen solubility, potentially leading to hypoxia, particularly in tissue preparations *in vitro* where oxygenation may already be marginal (Hajos and Mody, 2009). Hypoxia-induced injury and seizures are well-described phenomena, especially in the developing brain (Jensen and Wang, 1996; Sanchez and Jensen, 2005). One concern might be that the effects of hypoxia confound the results we described here. However, the time-course of the effects of hypoxia vs. hyperthermia are distinctly different. Membrane depolarization does not occur until about 5 min after the start of acute hypoxia (Jiang and Haddad, 1992; Bhave et al., 2003; Richard et al., 2010). We found that hyperthermia induces membrane depolarization within 1 min of exposure, however. Additionally, hypoxia-induced changes such as spreading depression and seizures more strongly affect CA1 than CA3 (Kawasaki et al., 1990; Kreisman et al., 2000; Sanchez and Jensen, 2005). In contrast, we found that hyperthermia more strongly influenced neurons in the CA3 area. We minimized effects of hypoxia by keeping superfusion rates high and measuring neuronal effects only during the first few minutes at high temperature, but we cannot entirely eliminate the possibility that hypoxia contributed to our results. We note that many neurons did recover fully after a test period of hyperthermia.

GIN cells belong to the class of somatostatin-expressing inhibitory interneurons that have been implicated in several aspects of epilepsy. Some studies have suggested that activation of somatostatin-positive interneurons, and the resulting release of somatostatin, have antiepileptogenic effects (Tallent and Siggins, 1997, 1999; Vezzani and Hoyer, 1999; Tallent and Qiu, 2008). However, somatostatin knockout mice were only mildly more prone to kainic acid-induced seizures than controls (Buckmaster et al., 2002). Although GIN interneurons increased their firing rates in response to hyperthermia, their activity did not reduce the firing of CA3 pyramidal cells. Further experiments are needed to explore whether somatostatin release itself has an effect on excitability under febrile conditions. Although decreased IPSCs were noted in response to hyperthermia (Qu et al., 2007; Qu and Leung, 2008, 2009), direct interneuron recordings were lacking. Both increases and decreases in inhibition may increase overall network excitability via inhibitory-inhibitory interactions. Investigations of inhibitory-inhibitory connections would provide further insight into the hyperthermia-driven circuit dynamics.

Gap junctions can synchronize interneurons very effectively (Galarreta and Hestrin, 1999; Gibson et al., 1999; Deans et al., 2001). In the hippocampus, neuronal gap junctions have been implicated in the generation and maintenance of gamma oscillations (Hormuzdi et al., 2001; Buhl et al., 2003), and in various forms of epileptogenesis (Carlen et al., 2000; Perez Velazquez and Carlen, 2000; Nemani and Binder, 2005; Gajda et al., 2006). However, the role of interneuronal gap junctions in seizure discharges has not been directly tested in the hippocampus. Gap junctions can be modulated by pH and hypoxia (Peracchia, 2004; Gonzalez-Nieto et al., 2008; Talhouk et al., 2008), but the effects of hyperthermic temperatures on neuronal gap junctions have not been investigated. We found that electrical coupling between GIN cells was maintained at hyperthermic temperatures. It may be that the role of gap junctions becomes more important for network synchrony during hyperthermic seizures when chemical transmission appears less effective.

Our results show that hyperthermia significantly increases the intrinsic excitability and spontaneous firing rates of neurons. This alone might lead to the generation of febrile seizures. CA3 neurons were most susceptible to heat, suggesting that the CA3 area is a likely site for the initiation of febrile seizures. To better understand febrile seizure mechanisms and treatment, it will be important to determine why some neuron populations are more sensitive to hyperthermia than others. Temperature-induced changes in excitability may also have beneficial effects. Anecdotal and epidemiological evidence suggests that fevers leading to body temperatures of 1.5–2.5°C above normal can acutely improve the behavior of children with autism spectrum disorders (Cotterill, 1985; Torres, 2003; Curran et al., 2007). The mechanisms of this effect are entirely unknown and indeed may not involve temperature changes directly (Mehler and Purpura, 2009). Nevertheless, it would not be surprising if the potent effects of modestly high temperatures on neuronal excitability cause significant changes in behavior.

### Conflict of interest statement

The authors declare that the research was conducted in the absence of any commercial or financial relationships that could be construed as a potential conflict of interest.
